# Modification of Polylactide Nonwovens with Carbon Nanotubes and Ladder Poly(silsesquioxane)

**DOI:** 10.3390/molecules26051353

**Published:** 2021-03-03

**Authors:** Mariia Svyntkivska, Tomasz Makowski, Ewa Piorkowska, Marek Brzezinski, Agata Herc, Anna Kowalewska

**Affiliations:** Centre of Molecular and Macromolecular Studies Polish Academy of Sciences, Sienkiewicza 112, 90-363 Lodz, Poland; mariiasv@cbmm.lodz.pl (M.S.); epiorkow@cbmm.lodz.pl (E.P.); mbrzezin@cbmm.lodz.pl (M.B.); asherc@cbmm.lodz.pl (A.H.); anko@cbmm.lodz.pl (A.K.)

**Keywords:** polylactide nonwovens, electrospinning, reinforced fibers, MWCNT, linear ladder-like poly(silsesquioxane)

## Abstract

Electrospun nonwovens of poly(L-lactide) (PLLA) modified with multiwall carbon nanotubes (MWCNT) and linear ladder-like poly(silsesquioxane) with methoxycarbonyl side groups (LPSQ-COOMe) were obtained. MWCNT and LPSQ-COOMe were added to the polymer solution before the electrospinning. In addition, nonwovens of PLLA grafted to modified MWCNT were electrospun. All modified nonwovens exhibited higher tensile strength than the neat PLA nonwoven. The addition of 10 wt.% of LPSQ-COOMe and 0.1 wt.% of MWCNT to PLLA increased the tensile strength of the nonwovens 2.4 times, improving also the elongation at the maximum stress.

## 1. Introduction

Electrospinning is a widely used technique to produce polymer fibers with different diameters and morphology. The nonwovens can be obtained via melt electrospinning or solution electrospinning [1]. The solution electrospinning does not require high temperature. Diameters and morphology of the electrospun fibers are determined by the type and molar mass of polymers, dispersity, and processing parameters, including applied voltage, tip-collector distance, type of solvent, feed rate, solution concentration, viscosity and conductivity. A wide range of electrospun polymer nonwovens has been obtained and studied for application in diverse fields: air [2] and water filtration [3], food packaging [4], tissue engineering [5] and reconstructive medicine [6], drug delivery systems [7], and antibacterial materials [8,9]. The drawback hindering the practical use of electrospun mats in some applications, for instance, filtration, is their poor tensile strength, as compared to woven fabrics. Fibers in nonwovens are loosely packed and weakly connected. The improvement of the mechanical performance of nonwovens can be achieved by modification of the fibers. Owing to their outstanding properties, including extremely high tensile strength and elastic modulus [10,11,12], carbon nanotubes (CNT) are attractive nanofillers for polymers [13]. The enhancement of properties of electrospun nonwovens by the introduction of CNT to electrospun polymer solutions was reported for different polymers, including poly(ethylene terephthalate), poly(vinylpyrrolidone), polyacrylonitrile/poly(vinyl chloride) blend, polyamide 6, and polylactide (PLA), among others [3,14,15,16,17,18,19,20,21]. Modification of electrospun nonwovens can be also achieved by incorporating other nanoparticles, for example, graphene oxide [14], cellulose nanocrystals [22], or hydroxyapatite nanoparticles [23].

PLA, a biodegradable polyester having good mechanical properties, is the most promising biobased polymer for replacement of conventional thermoplastics [24]. PLA’s ability to crystallize depends on its enantiomeric composition; with the increasing content of units of different chirality, the ability to crystallize worsens. Slowly crystallizing PLAs can be cooled to the glassy state and crystallized during subsequent heating via “cold crystallization”. The effective way to accelerate the crystallization of PLA is the addition of nucleating agents [25]. Additionally, shearing of PLA melt can enhance its crystallization [26,27]. To broaden its applications, PLA’s properties are modified by various routes, including copolymerization, chain extension, plasticization, blending with other polymers, fillers, nanofillers, and fibers [28,29,30,31,32,33,34,35,36,37]. PLA modification with oligomeric linear ladder poly(silsesquioxane)s (LPSQ-R) with different sides substituents, including methoxycarbonyl (R = COOMe) groups was described recently by Herc et al. [38]. LPSQ-R are well-defined macromolecules with a double chain siloxane backbone, which makes them more rigid than typical polysiloxanes and limits their coiling in solutions. Interestingly, a significant increase of elongation at break was achieved for PLA blends with 5 wt.% of LPSQ-COOMe, with only a minor decrease of the yield strength, as compared to neat PLA. LPSQ-COOMe described in this report is a viscous amorphous substance of good thermal stability, well soluble in organic solvents. Herc et al. [39] recently demonstrated that LPSQ-COOMe enhanced the thermal stability of PLA. 

Electrospinning allowed to obtain PLA fibers with diameters in the nanometer [40,41] and micrometer range [42,43,44]. To obtain modified PLA nonwovens, electrospinning of the polymer solutions with added plasticizers, nanofillers, or antibacterial substances, among others, was carried out, although post-electrospinning modification of fibers was also reported. For example, antibacterial activity of PLA nonwovens was achieved by introducing antibacterial agents to the solutions [8,9,42]. Plasticization of PLA/poly(hydroxybutyrate) blend nonwovens with oligomeric lactic acid (OLA) and acetyl(tributyl citrate) was described by Arriata et al. [45,46]. Leones et al. [47] used OLA to plasticize PLA nonwovens. In turn, the improvement of tensile strength of electrospun PLA mats was achieved by addition of nanofillers; for example, by cellulose nanocrystals [48], hydroxyapatite nanorods and graphene oxide [49]. PLA composite nonwovens with 5 wt.% of siliceous sponge spicules exhibited a tensile strength four times higher than that of neat PLA material [50].

The addition of nanocarbon materials to electrospun PLA solutions can result in an improvement of the tensile strength and elastic modulus [14,47,48,51]. A fourfold increase of the tensile strength and elastic modulus was reported for PLA nonwovens with 3 wt.% of multiwall CNT (MWCNT) relative to those of neat PLA nonwovens, although accompanied by a significant decrease of elongation at break [48]. Similar enhancement of the tensile strength of PLA nonwovens was achieved through the addition of 1 wt.% of functionalized MWCNT [51]. 

In this study, LPSQ-COOMe and MWCNT were used for modification of poly(L-lactide) (PLLA) electrospun nonwovens. PLLA grafted on MWCNT was prepared and used for electrospinning; MWCNT content was 0.1 wt.%. For comparison, nonwovens of commercial PLLA with the same content of MWCNT were also obtained. In addition, fibers of PLLA with 5 and 10 wt.% of LPSQ-COOMe were prepared, and also fibers with the same contents of LPSQ-COOMe and 0.1 wt.% of MWCNT with respect to PLLA. LPSQ-COOMe is not biodegradable, but like other silsesquioxanes [52] it is non-toxic and biocompatible. The influence of the additives on the morphology and diameters of the nonwovens as well as on their thermal and mechanical properties was studied. It was demonstrated that the modification with a small amount of MWCNT and 5–10 wt.% of LPSQ-COOMe significantly improved the tensile strength of the nonwovens. It is also worth noting that LPSQ-COOMe also improved thermal stability of the studied nonwovens.

## 2. Materials and Methods 

### 2.1. Materials

Commercial PLLA Luminy^®^ L175 was obtained from Total Corbion (The Netherlands) with D-lactide content below 1%, according to the supplier. Weight average molar mass M_w_ of 78 kg/mol and dispersity M_w_/M_n_ of 1.8 were measured by size exclusion chromatography (SEC) with a multi-angle laser light scattering detector in dichloromethane using an Agilent Pump 1100 Series (preceded by an Agilent G1379A Degasser), equipped with a set of two PLGel 5 µ MIXED-C columns. A Wyatt Optilab Rex differential refractometer and a Dawn Eos (Wyatt Technology Corporation) laser photometer were used as detectors. Dichloromethane was used as eluent at a flow rate of 0.8 mL min^-1^ at room temperature (RT). 

MWCNT Nanocyl NC-7000 (Nanocyl, Belgium) with an average diameter of 9.5 nm, length of 1.5 μm, and 90% purity, were used. PLLA covalently attached to MWCNT (PL-g-CNT) was synthesized by ring-opening polymerization of L,L-lactide according to a well-established procedure [53,54]. LPSQ-COOMe was synthesized as previously described [38,39]. Procedures for the synthesis of PL-g-CNT and LPSQ-COOMe are described below.

Dichloromethane purchased from StanLab (Poland) (purity 99.5%) and acetone purchased from POCH (Poland) (purity 99%) were used as received.

### 2.2. Synthesis of PL-g-CNT

Ring-opening polymerization of L,L-lactide was carried out as follows: Sn(Oct)_2_ (1 mL of 0.25 mol L^−1^ solution in dry tetrahydrofuran (THF)) and L,L-Lactide (L,L-LA, 7.42 g, 51.5 mmol) were transferred under vacuum into break-seals and then sealed after being frozen in liquid N_2_. MWCNT−OH (7.4 mg) was placed directly into the reaction ampule, dried under vacuum for 4 h, and sealed. Break-seals that contained the Sn(Oct)_2_/THF solution and L,L-LA monomer and a tube that contained dry MWCNTs in a vial were sealed to the glass reaction vessel (∼10 mL). Under vacuum, the break-seals and vial were broken, and all components were mixed at RT. THF was removed by distillation, and then the reaction vessel was sealed. The reaction vessel was placed into an ultrasonic bath for 60 min (130°C) to disperse MWCNT−OH, then placed into a thermostated oil bath, and the polymerization was carried out at 130 °C for approx. 24 h. The resulting polymer was dissolved in CHCl_3_ and precipitated into methanol, separated by filtration, and washed several times with methanol. The mass of the vacuum-dried product was 6.31 g (85% yield). The content of MWCNTs in the product was 0.1 wt.%. The molecular characteristics of the product were determined according to the procedure described previously [53]. It was found that PL-g-CNT was a mixture composed of PLLA grafted on MWCNT and free PLLA. Only approximately 15−20 wt.% of the PLLA chains were found to be polymerized from the MWCNT. M_w_ and M_w_/M_n_ of the free PLLA fraction were 82 kg/mol and 1.4, respectively, as determined by SEC using the equipment described above. The molar masses of both PLLA fractions should be similar [55]. ^1^H NMR of PL-g-CNT (CDCl_3_): δ= 5.16 (q, 1H, CH-CH_3_ polymer), 1.67 (d, 3H, CH_3_ polymer) ppm. 

### 2.3. Synthesis of LPSQ-COOMe

LPSQ-COOMe was synthesized as previously described [38,39]. Methyl thioglycolate (Acros Organics, 95%) was added into a solution of ladder-like poly(vinylsilsesquioxanes), M_n_ = 1000 g/mol, M_w_/M_n_ = 1.4, obtained as described in [56], dissolved in dry THF (c = 8 wt.%) and placed in a quartz crucible. The ratio of reagents was [HS]_0_/[Vi]_0_ = 1.4). A photoinitiator (2,2-dimethoxy-2-phenylacetophenone, Acros Organics, 99%) was then added with stirring to the solution of reagents at [Vi]_0_/[DMPA]_0_ = 50. The mixture was irradiated for 15 min with UV light (λ = 365 nm). Volatiles were removed under reduced pressure and the residue was purified by precipitation into a large amount of hexane/ethyl acetate (1: 1 *v*/*v*). The product was dried under high vacuum at RT to the constant weight (reaction yield 91%) and characterized with ^1^H NMR spectroscopy using a Bruker DRX-500 MHz spectrometer: δ [ppm] (THF-d8): 0.16 (s. OSiMe_3_), 1.06 (m. SiCH_2_), 2.77 (m. CH_2_S), 3.25 (m. SCH_2_), 3.65 (s. OCH_3_). The ^1^H NMR spectrum is shown in Figure S1 of Supporting Information (SI). Full NMR characteristics have been previously published in [38,39].

The obtained LPSQ-COOMe was essentially an amorphous substance with T_g_ at −41.2 °C as determined by DSC during heating at 10 °C /min from −100 °C.

### 2.4. Preparation of Nonwovens

Before further use, commercial PLLA pellets were dried under reduced pressure for 4 h at 100 °C. After drying, PLLA was dissolved in dichloromethane to obtain 12 wt.% solution. LPSQ-COOMe was added to PLLA solution to obtain 5:100 and 10:100 weight ratios of the modifier to PLLA (PL-LPSQ5, PL-LPSQ10). Furthermore, MWCNT were added to the solution containing neat PLLA and PLLA with LPSQ-COOMe to obtain 1:1000 weight ratio of MWCNT to PLLA (PL-CNT, PL-LPSQ5-CNT, PL-LPSQ10-CNT). All the solutions and dispersions were mixed with acetone in a volume ratio of 7:3. The dispersions were homogenized using an ultrasonic homogenizer, the Hielscher UP 200S (Hielscher, 130 Germany, power 200 W, amplitude 30%, frequency 24 kHz), at RT for 20 min.

PL-g-CNT was dissolved in dichloromethane to obtain a 6 wt.% solution. Then the solution was mixed with acetone in a volume ratio of 7:3 and homogenized, as described above. 

PLLA-based nonwovens were electrospun at RT and relative humidity of 35 –40% using a system consisting of a high voltage power supply CM5 (Simco-Ion, The Netherlands) and an aluminum plate 8 × 8 cm as a collector. Glass syringes and a step motor, model T-LLS105 from Zaber (Canada), were used for dosing of solutions. The tip-collector distance was 25 cm in all cases. The parameters of the electrospinning are listed in Table 1. The polymer concentrations in the solvent and other parameters of the electrospinning were selected based on preliminary experiments. The preparation scheme of the nonwovens is shown in Figure S2 in SI.

The obtained nonwovens were dried for 4 h at 35 °C under reduced pressure and then stored in a desiccator at RT. All measurements were carried out at least two days after the electrospinning.

### 2.5. Characterization

The thicknesses of all nonwovens were measured and their surface densities were determined by weighing. The surfaces of the nonwovens were examined using scanning electron microscopy (SEM) with energy dispersive spectroscopy (EDS) JEOL 6010LA (JEOL, Japan) at an accelerating voltage of 10 kV. Before the examination, the surfaces were vacuum sputtered with a 10 nm gold layer using a coater (Quorum EMS150R ES, UK). Diameter distributions of the fibers were determined from SEM micrographs. 

Water contact angles (WCA) of 5 µl distilled water droplets with the nonwovens were determined at RT, using a goniometer 100-00-230 NRL Rame Hart (USA) with the Image Drop Analysis program. In each case, the WCA measurements were carried out five times and average values were calculated. 

Thermal properties of the nonwovens were studied using differential scanning calorimetry (DSC 3 Mettler Toledo, Switzerland) during heating at 10 °C/min. Thermogravimetric analysis was performed using the TGA 550 from TA instruments (USA) in a nitrogen atmosphere at a heating rate of 20 °C/min.

Tensile drawing of nonwoven specimens was performed with the Linkam TST 350 Minitester (UK) at 25 °C. Three 10 mm wide strips of each material were drawn, with a distance between grips of 20 mm, at a rate of 2 mm/min (10%/min). The tests were carried out at least 48 h after preparation of the nonwovens. 

Selected PLLA-based nonwovens were analyzed with Fourier-transform infrared spectroscopy (FTIR) using a Thermo Scientific Nicolet 6700 FT-IR instrument with an ATR (Attenuated Total Reflectance) (USA). FTIR spectra in the range of 500–4000 cm^−1^ were recorded with a 1 cm^−1^ resolution.

## 3. Results and Discussion

**Figure 1:**
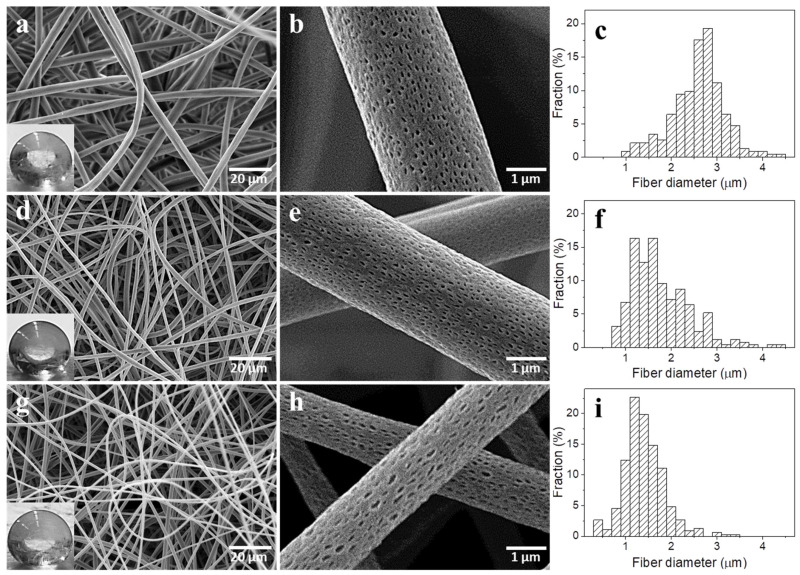
SEM micrographs of nonwovens: PLLA (**a,b**), PL-CNT (**d,e**), PL-g-CNT (**g,h**) and fiber diameter distributions; PLLA (**c**), PL-CNT (**f**), PL-g-CNT (**i**). Water drops on nonwoven surfaces are shown in the insets.

**Figure 2:**
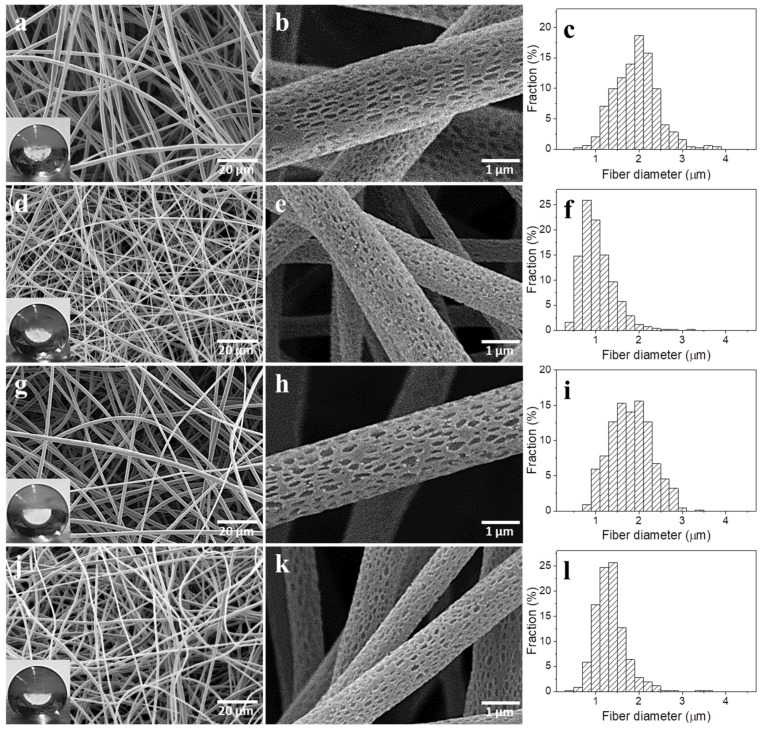
SEM micrographs of nonwovens: PL-LPSQ5 (**a,b**), PL-LPSQ5-CNT (**d,e**), PL-LPSQ10 (**g,h**), PL-LPSQ10-CNT (**j,k**) and fiber diameter distributions: PL-LPSQ5 (**c**), PL-LPSQ5-CNT (**f**), PL-LPSQ10 (**i**), PL-LPSQ10-CNT (**l**). Water drops on nonwoven surfaces are shown in the insets.

The structure of the electrospun PLLA-based nonwovens, fiber morphology, and the fiber size distributions are shown in Figure 1 and Figure 2, whereas parameters of the nonwovens are collected in Table 2. All nonwovens were composed of uniform and defect-free fibers, randomly distributed. It is worth noting that TGA thermograms, shown in Figure S3 in SI, evidence that the solvents evaporated during the electrospinning and post-electrospinning drying; weight loss at 100 °C was below 0.1% in each case. Moreover, the TGA thermograms in Figure S3 in SI and data collected in Table S1 in SI show that all the materials with LPSQ-COOMe exhibited better thermal stability in comparison to neat PLLA, as already reported [39]. 

Diameters of a majority of neat PLLA fibers were in the 1.5–3.5 µm range, with an average of 2.5 µm, whereas diameters of PL-CNT and PL-g-CNT were smaller, mostly below 2 µm, with an average of 1.7 and 1.8 µm, respectively. The decrease of the PL-CNT fiber diameters was caused by an increase in the electrical conductivity of the solution. As a result, the solution underwent a greater extension under the influence of the electrostatic field, which caused the reduction of fiber diameters [6,57]. The same applies to PL-g-CNT electrospinning process, although it is worth noting that PL-g-CNT concentration in the solution had to be decreased in comparison to that of PL-CNT because of increased viscosity due to grafting of polymer chains on MWCNT. The pores on the fiber surfaces resemble those observed in [44,58] due to the breath figure mechanism. As a result of the evaporative cooling of the solvent, water vapor condensed and droplets formed on the surface of the fiber jets, causing the formation of pores. Moreover, the pores were elongated in the directions parallel to the fiber axes, as a result of deformation during jet stretching. 

The addition of 5 and 10 wt.% of LPSQ-COOMe to PLLA resulted in thinner fibers, mostly with diameters ranging from 1 to 3 µm, and average diameters of 1.8 and 1.7 µm, respectively, as shown in Figure 2 and Table 2. The decreased average fibers diameters of PL-LPSQ5 and PL-LPSQ10 as compared to those of PLLA could result from a decrease of solution viscosity resulting from the presence of LPSQ-COOMe. Fibers of PL-LPSQ5-CNT and PL-LPSQ10-CNT were even thinner, mostly with diameters ranging from 0.5 to 1.5 µm and from 1 to 2 µm, with average values of 0.9 and 1.2 µm, respectively. The nonwoven thickness ranged from 0.68 to 0.84 mm. The thickest was the nonwoven of neat PLLA, whereas the thinnest were the nonwovens with MWCNT, composed of the markedly thinner fibers.

Figure 1 and Figure 2 show also water droplets placed on the nonwoven surfaces, whereas the WCA values are listed in Table 2. All the obtained electrospun PLLA-based nonwovens were hydrophobic, with WCA values close to 155°. It is known that hydrophobicity of nonwovens results from their surface roughness and air entrapped between fibers [59].

**Figure 3:**
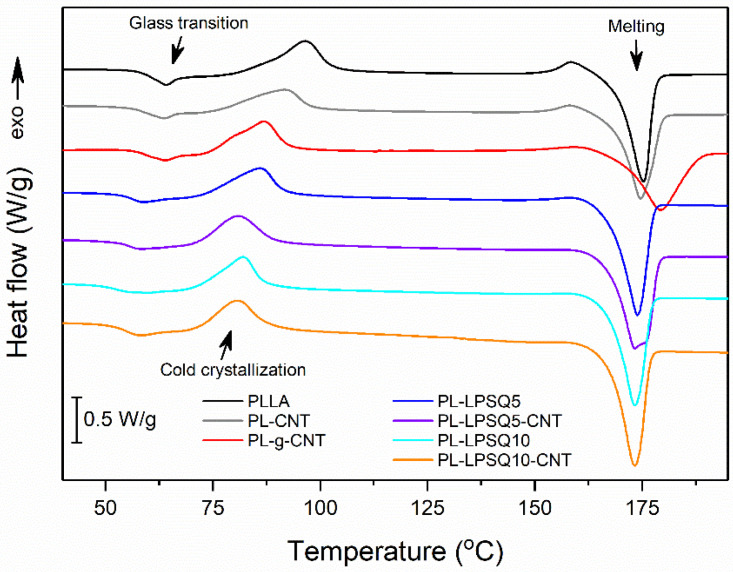
DSC heating thermograms of PLLA-based nonwovens.

DSC heating thermograms of PLLA-based nonwovens are compared in Figure 3, whereas the calorimetric parameters are collected in Table 3. Each thermogram shows a glass transition, a cold crystallization exotherms, and a melting endotherm. In addition, on all thermograms, pre-melting exotherms, attributed to the transition from the disordered orthorhombic alpha’ form to the ordered orthorhombic alpha form [60], are visible, with a maximum at 157–159 °C. The cold crystallization exotherms, especially those of the materials with LPSQ-COOMe, exhibit long high-temperature tails, overlapping the pre-melting recrystallization exotherms, hence the latter are less pronounced and their enthalpies listed in Table 3 are only approximate. However, in each case the sum of enthalpies of the exothermic effects was equal to the melting enthalpy, evidencing that the nonwovens were amorphous before heating in DSC. T_g_ of neat PLLA nonwoven was at 59 °C, whereas the cold crystallization peak (T_cc_) was at 97 °C. T_g_s of both PL-CNT and PL-g-CNT were nearly the same, at 58 °C. T_g_s of the nonwovens of PLLA blends with 5 and 10 wt.% of LPSQ-COOMe were lower, at 55 and 52–53 °C, respectively, due to the plasticizing effect of the additive. However, taking into account the T_g_s of PLLA, LPSQ-COOMe and PLLA blends with LPSQ-COOMe, it can be calculated, using the Fox equation, that in PLLA with 5 and 10 wt.% of LPSQ-COOMe about 3 and 5 wt.% of the additive, respectively, is dispersed on a molecular level. This is in accordance with ref. [38], where phase separation was observed in PLA blends with LPSQ-COOMe. Figure S4 in SI shows SEM EDS mapping of silicon in PL-LPSQ5 and PL-LPSQ10 that confirms the presence of LPSQ-COOMe and its dispersion in the PLLA matrix. Inclusions of the additive were not discernible, most probably being too small, as in ref. [38]. It is also observed that T_cc_ of composite materials decreased in comparison to that of neat PLLA nonwoven, to 92 °C for PL-CNT and even more, to 87 °C, for PL-g-CNT. T_cc_s of PL-LPSQ5 and PL-LPSQ10 nonwovens were even lower, at 86 °C and 82 °C, respectively. The presence of CNT further enhanced the decrease of T_cc_, to 81 °C for both PL-LPSQ5-CNT and PL-LPSQ10-CNT. In turn, the melting peak temperatures (T_m_) of the materials were at 173–175 °C. The only exception was PL-g-CNT, whose T_m_ of 179 °C was higher than that of PL-CNT, indicating the melting of thicker crystals, despite its low T_cc_. CNT are known to nucleate crystallization of PLA [61], whereas LPSQ-COOMe increases the mobility of PLA chains and also enhances its cold crystallization [38]. It is worth noting that PLLA in PL-g-CNT was optically pure. Moreover, the additives can enhance the orientation of PLLA in the fibers during electrospinning, which also can promote cold-crystallization of the polymer.

**Figure 4:**
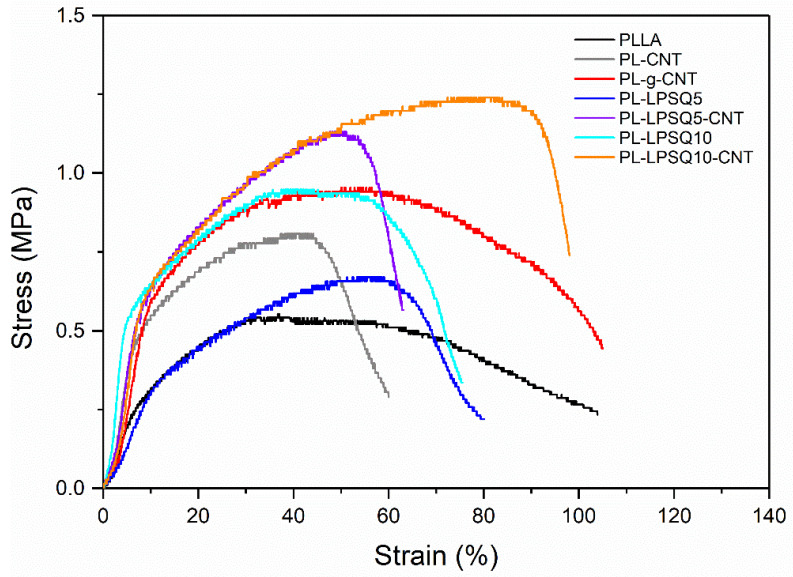
Stress-strain dependencies of PLLA-based nonwovens.

Figure 4 shows the tensile behavior of the nonwovens, whereas the mechanical parameters are collected in Table 4. Fibers in the nonwovens were randomly distributed, without any preferred orientation. When the nonwovens were strained, the fibers tended to orient parallel to the stretching direction. Loosely connected structure without strong bonds between the fibers at their cross points facilitated the fiber alignment during drawing. However, some fibers broke at early stages of drawing, thus decreasing the tensile stress. In Figure 4 the engineering stress, calculated as a ratio of force to initial cross-section area of the nonwoven, is plotted vs. engineering strain. It is seen that with increasing strain the stress increases, passes through a maximum, and decreases, less or more sharply. The maximum stress value recorded during the drawing of neat PLLA nonwoven was 0.5 MPa. Similar values measured for electrospun neat PLA nonwovens were reported in [49,62]. The presence of 0.1 wt.% of CNT increased significantly the tensile strength to 0.8 and 0.95 MPa for PL-CNT and PL-g-CNT, respectively. The effect of 5 wt.% of LPSQ-COOMe on the PLLA nonwoven strength was weak, but PL-LPSQ10 exhibited a strength of 0.95 MPa. However, the highest strength was achieved for PL-LPSQ5-CNT and PL-LPSQ10-CNT, 1.1 and 1.2 MPa, which exceeded more than two times the strength of neat PLLA nonwoven. It is worth noting that the maximum stress of PL-LPSQ10-CNT was achieved at an elongation of approx. 80%, significantly larger than in the case of the other materials.

CNT-containing materials are known to exhibit outstanding mechanical properties, due to stress transfer from the weaker polymer to the stronger nanofiller, which allows a higher loading to be achieved, thus the improvement in strength [14]. However, in the nonwovens studied the weight ratio of MWCNT to PLLA was only 1:1000, therefore we attribute the increase in strength also to the enhancement of PLLA chain orientation during electrospinning. An increase in electrical conductivity of the solution due to the presence of CNT results in a greater extension under the influence of the electrostatic field, which can promote not only the fiber diameter reduction but also the orientation of polymer chains. It must be noted that CNT are highly anisotropic particles, which tend to be oriented during fiber jet stretching. It is also worth noting that the effect of LPSQ-COOMe on the tensile properties of the PLLA nonwovens was different than on PLA films, whereas at 5 wt.% content of the additive a large elongation at break, 230%, was achieved, accompanied by a minor, approx. 10%, decrease of the tensile strength [38]. LPSQ-COOMe double-strand structure hampers coiling, and orientation of its macromolecules during electrospinning can also promote the orientation of PLLA chains, especially that they are capable of supramolecular interactions with PLLA. Side ester groups of LPSQ-COOMe may participate in weak C-H⋯O=C hydrogen bonds, analogously to those in stereocomplex structures formed between enantiomeric chains of PLLA and poly(D-lactide) [63], and postulated for hybrid stereocomplex-PLA/LPSQ-COOMe blends [39]. The enhanced PLLA orientation can contribute to the improved strength of the modified nonwovens.

**Figure 5:**
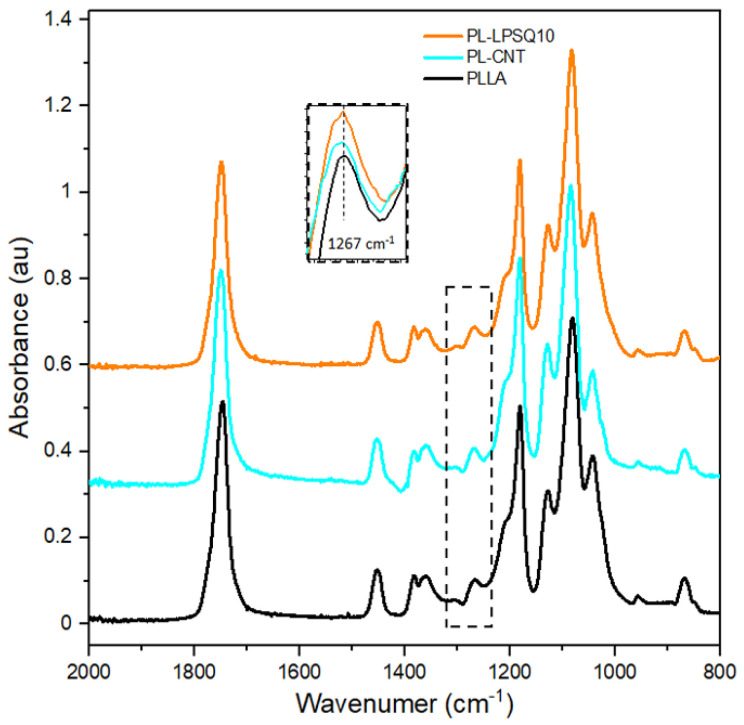
FTIR spectra of PLLAbased nonwovens in 2000–800 cm^−1^ range.

Figure 5 shows FTIR spectra of the selected nonwovens positioned perpendicular to the beam in the 2000–800 cm^−1^ range with the inset showing the enlarged peak at 1267 cm^−1^.

FTIR spectra of the selected nonwovens, shown in Figure 5, are typical for amorphous PLLA. 10 wt.% content of LPSQ-COOMe in PLLA corresponds to approx. 3 mol% content of functional groups, therefore the LPSQ-COOMe bands did not show up in the spectra, especially that some of them, e.g., those related to C=O or CH_3_ groups, are overlapping with PLLA bands [39]. The band near 1750 cm^−1^ corresponds to C=O stretching, whereas the bands of the CH_3_ asymmetric and symmetric bending and the first overtone of CH bending are seen near 1450, 1380, and 1360 cm^−1^ [64]. The bands near 1180, 1130, 1080, 1040 and 870 cm^−1^ are attributed to the asymmetric COC stretching and the asymmetric CH_3_ rocking, the CH_3_ symmetric rocking, the COC symmetric stretching, the C-CH_3_ stretching, and the C-COO stretching, respectively [64]. The band near 1270 cm^−1^ is attributed to the CH bending and COC stretching. The spectra of the PLLA-based nonwovens shown in Figure 5 were nearly identical except for the 1266–1267 cm^−1^ band. The peaks were significantly higher for the modified PLLA nonwovens than for the neat PLLA nonwoven, as shown in the inset in Figure 5. This band is sensitive to the presence of gauche–gauche (gg) conformers in the PLLA chain, which are less energy-favorable than the gauche–trans (gt) conformers [65]. It was recently demonstrated that gg conformers can originate from the stretching of the PLLA macromolecule [66]. Thus, the increased intensity of 1266–1267 cm^−1^ band may evidence enhanced stretching and orientation of PLLA chains in the modified PLLA fibers, which contributed to the increased tensile strength of the modified nonwovens.

## 4. Conclusions

PLLA-based nonwovens were obtained by electrospinning. The PLLA nonwovens were modified with 0.1 wt.% of multiwall carbon nanotubes (MWCNT) and 5 and 10 wt.% of linear ladder poly(silsesquioxane) with methoxycarbonyl side groups (LPSQ-COOMe), which were added to the polymer solution before the electrospinning. In addition, nonwovens of PLLA grafted to modified MWCNT were electrospun; the content of MWCNT was 0.1 wt.%. The additives significantly reduced the fiber diameters. An average diameter decreased from 2.5 µm for neat PLLA fibers to about 1 µm for PL-LPSQ5-CNT and PL-LPSQ10-CNT. LPSQ-COOMe decreased T_g_ of the fibers by up to 7 °C and increased their thermal stability. A decrease of cold crystallization temperature of modified fibers in comparison to those of neat PLLA was observed; the cold crystallization peak shifted from 97 °C to 81 °C. All modified nonwovens exhibited higher tensile strength than the neat PLLA nonwoven. The addition of 10 wt.% LPSQ-COOMe and 0.1 wt.% of MWCNT to PLLA increased the tensile strength of the nonwovens 2.4 times, improving also the elongation at maximum stress. FTIR analysis suggested the stronger orientation of PLLA chains in the modified fibers, which contributed to the improvement of their tensile strength.

## Figures and Tables

**Table 1:** Parameters of electrospinning.

Sample Code	Flow Rate (mL/min)	Voltage (kV)
PLLA	0.065	20
PL-LPSQ5	0.065	20
PL-LPSQ10	0.065	20
PL-g-CNT	0.065	18
PL-CNT	0.052	18
PL-LPSQ5-CNT	0.052	18
PL-LPSQ10-CNT	0.052	18

**Table 2:** Characteristics of PLLA-based nonwovens: thickness, surface density, average fiber diameter, the most probable diameter (at the histogram maximum) and water contact angle (WCA).

Sample Code	Thickness (mm)	Surface Density (mg/cm²)	Average Fiber Diameter (µm)	Most Probable Fiber Diameter (µm)	WCA (°)
PLLA	0.84	6.8	2.5	2.7–2.9	155° ± 3
PL-CNT	0.58	5.9	1.7	1.5–1.7	154° ± 4
PL-g-CNT	0.68	6.0	1.8	1.1–1.3	156° ± 2
PL-LPSQ5	0.74	7.1	1.8	1.9–2.1	156° ± 2
PL-LPSQ5-CNT	0.68	6.3	0.9	0.7–0.9	155° ± 4
PL-LPSQ10	0.75	8.1	1.7	1.9–2.1	156° ± 3
PL-LPSQ10-CNT	0.68	7.0	1.2	1.2–1.4	156° ± 3

**Table 3:** Calorimetric parameters of PLLA-based nonwovens: T_g_–the glass transition temperature, T_cc_, H_cc_–the cold crystallization peak temperature and enthalpy, respectively, T_m_ and H_m_–the melting peak and enthalpy, respectively, the enthalpy of the pre-melting recrystallization is given in brackets.

Sample Code	T_g_ (°C)	T_cc_ (°C)	H_cc_ (J/g)	T_m_ (°C)	H_m_ (J/g)
PLLA	59	97	34 (6)	175	41
PL-CNT	58	92	33 (4)	175	39
PL-g-CNT	58	87	36 (4)	179	41
PL-LPSQ5	55	86	44 (4)	174	48
PL-LPSQ5-CNT	55	81	49 (2)	173	51
PL-LPSQ10	52	82	48 (2)	173	50
PL-LPSQ10-CNT	53	81	50 (2)	173	52

**Table 4:** Mechanical parameters of PLLA-based nonwovens.

Sample Code	Maximum Stress (MPa)	Strain at Maximum Stress (%)
PLLA	0.50	52
PL-CNT	0.81	40
PL-g-CNT	0.95	62
PL-LPSQ5	0.67	57
PL-LPSQ5-CNT	1.09	52
PL-LPSQ10	0.95	53
PL-LPSQ10-CNT	1.22	83

## Data Availability

The data presented in this study are available on request from the corresponding author.

## Supplementary Materials

Figure S1: ^1^H NMR spectrum of LPSQ-COOMe, recorded in THF-d_8_ on a Bruker 200 spectrometer. Figure S2: Preparation scheme of PLLA-based nonwovens. Figure S3: TGA thermograms of PLLA-based nonwovens, in a nitrogen atmosphere at a heating rate of 20 °C/min. Table S1: Thermogravimetric data of PLLA-based nonwovens. Figure S4: EDS carbon and silicon mapping of (**a,b**) PL-LPSQ5 and (**c,d**) PL-LPSQ10 nonwovens.
