# Supplementary material for: Modification of Polylactide Nonwovens with Carbon Nanotubes and Ladder Poly(silsesquioxane)
Source: Molecules. 2021 Mar 3;26(5):1353. doi: 10.3390/molecules26051353 (PMC7961909; doi:10.3390/molecules26051353)
Supplement: Supplementary file 1 [file molecules-26-01353-s001.pdf]

molecules

MDPI

Supplementary Information

Modification of polylactide nonwovens with carbon nanotubes and ladder poly(silsesquioxane)

Mariia Svyntkivska[1], Tomasz Makowski[1,*], Ewa Piorkowska[1], Marek Brzezinski[1], Agata Herc[1], Anna Kowalewska[1]

[1]Centre of Molecular and Macromolecular Studies Polish Academy of Sciences, Sienkiewicza 112, 90-363 Lodz, Poland

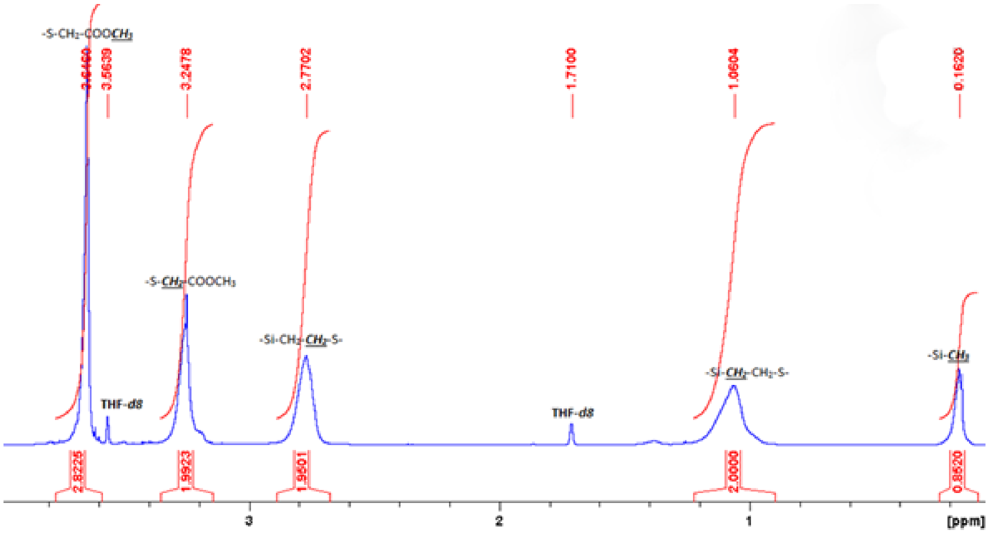

Figure S1. ^{1}H NMR spectrum of LPSQ-COOMe, recorded in THF-d_8 on a Bruker 200 spectrometer.

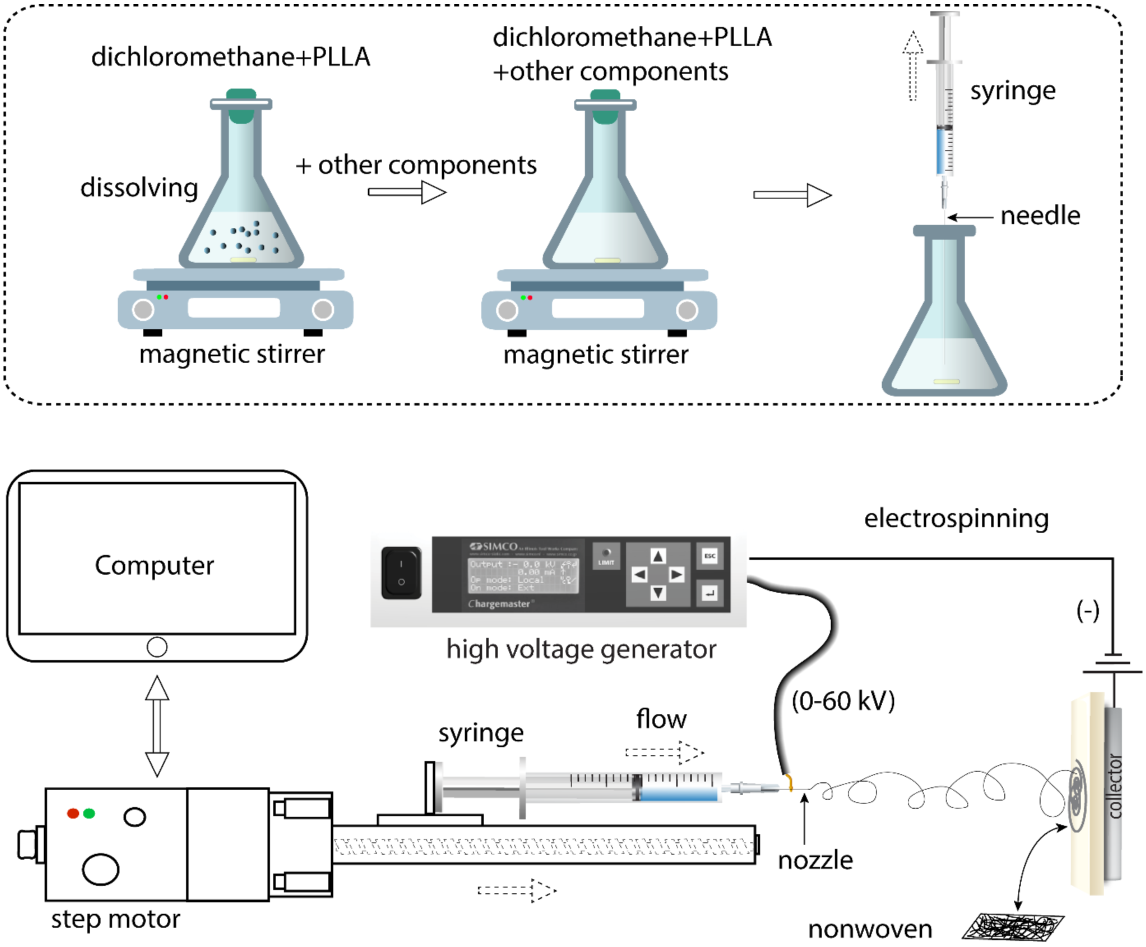

Figure S2. Preparation scheme of PLLA-based nonwovens.

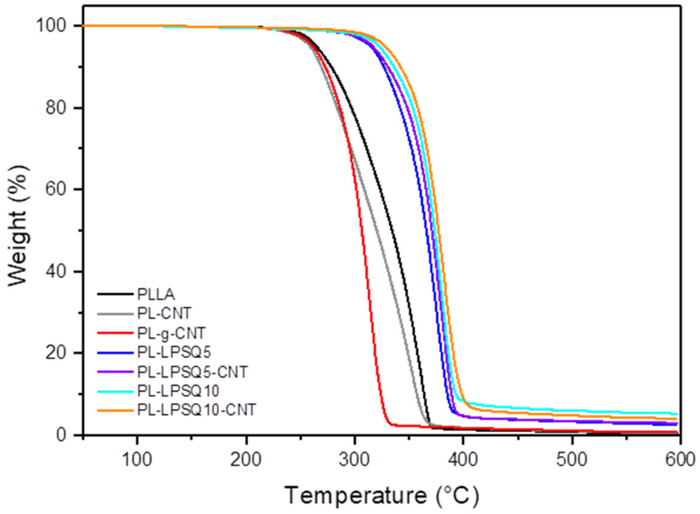

Figure S3. TGA thermograms of PLLA-based nonwovens in a nitrogen atmosphere at a heating rate of 20 °C/min.

Table S1. Thermogravimetric data of PLLA-based nonwovens: T_1 – 1% weight loss temperature; T_5 – 5% weight loss temperature, T_{10} – 10% weight loss temperature, T_d – peak temperature of weight loss derivative with respect to temperature.

Sample code	T_1 (°C)	T_5 (°C)	T_{10} (°C)	T_d (°C)
PLLA	238	265	278	365
PL-CNT	229	259	269	355
PL-g-CNT	232	261	272	314
PL-LPSQ5	265	314	327	374
PL-LPSQ5-CNT	264	316	330	377
PL-LPSQ10	260	322	336	380
PL-LPSQ10-CNT	281	328	342	382

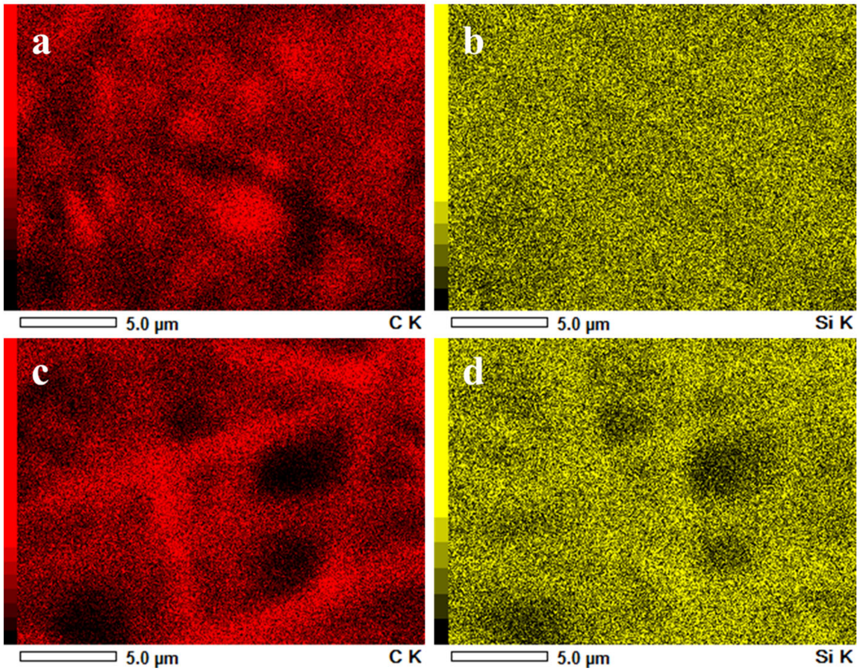

Figure S4. EDS carbon and silicon mapping of (**a**,**b**) PL-LPSQ5 and (**c**,**d**) PL-LPSQ10 nonwovens.
